# Perceptions of Contextual Stressors in Physical Education. A Qualitative Case Study

**DOI:** 10.3389/fspor.2020.528979

**Published:** 2020-10-09

**Authors:** Eli-Karin Sjåstad Åsebø, Helga S. Løvoll, Rune Johan Krumsvik

**Affiliations:** ^1^Faculty of Arts and Physical Education, Department of Physical Education, Volda University College, Volda, Norway; ^2^Department of Education, Faculty of Psychology, University of Bergen, Bergen, Norway

**Keywords:** physical education (PE), lower secondary school, stressors, students' experiences, case study

## Abstract

**Background:** Daily stressors have a significant impact on students' educational outcomes. However, research on students perceived and common contextual stressors in physical education (PE) lessons is limited.

**Purpose:** To identify potential contextual stressors in PE contexts and what students perceive as stressors.

**Participants:** Ninth-grade students (age 14-15) and their PE teachers recruited from three classes in one lower secondary school in Norway.

**Research Design:** This qualitative case study used data generated from descriptive field notes from participant observations in PE lessons, formal interviews and informal conversations with PE teachers, focus group and individual interviews with students, and a supplementary survey using the TurningPoint student response system. Conversations were transcribed verbatim and analyzed using reflexive thematic analysis (Braun and Clarke, 2006; Tolmie et al., 2011; Braun et al., 2019) and the NVivo 12 Pro analysis software. The survey was analyzed using IBM SPSS Statistics 21.

**Findings:** This study supports and expands previous research exploring students' stressors in PE and highlights the volume and variety of potential stressors in PE contexts. The findings shed light on certain similarities and differences that may exist between students of different genders and grades and with different past physical activity experiences. In the present study, spectators, in addition to difficult tasks and low self-efficacy, seemed particularly stressful for girls. This article presents nuances revealed by various qualitative approaches and a supplementary survey.

**Conclusion:** Students in this study experience a multitude of stressors during PE lessons. These include stressors in the teaching, physical, and social environments, as well as personal factors. The stressors experienced depend on the situation, the lesson content, the parties involved, students' past experiences, and their appraisal of these stressors. In our sample, girls seemed to be more vulnerable to contextual stressors in PE than boys.

## Introduction

This qualitative case study research (Stake, 2006) addresses an important part of current state of knowledge internationally, which we have limited research knowledge about in the Norwegian context; students' perceptions of contextual stressors in physical education (PE) lessons.

In addition to *Bildung* and reflection, one of the aims of PE is to contribute to public health goals and increase physical activity among young people (Norwegian Directorate for Education Training, 2015). In a national mapping study of PE (5th−10th grades) in Norway, it appears that most students like PE, but there is a small group that “dread” it (Moen et al., 2018). Additionally, they see a negative development from primary to lower secondary school, where students like PE less and experience a lack of mastery as they grow older. This tendency is most evident among girls. Although PE is a popular subject, some students in Norway experience a number of challenges, barriers, and stressors (Säfvenbom et al., 2015; Lyngstad et al., 2016; Walseth et al., 2017, 2018; Thorjussen and Sisjord, 2018; Røset et al., 2019). Similar experiences are observed in many other countries (Groves and Laws, 2000; Flintoff and Scraton, 2001; Hills, 2007; Redelius and Larsson, 2010; Fagrell et al., 2012; Cardinal et al., 2013; Fisette, 2013; Wiltshire et al., 2017; Martins et al., 2018; Munk and Agergaard, 2018; Domville et al., 2019; Joy and Larsson, 2019). Showering together in the locker room may also be challenging for some (O'Donovan et al., 2015; Johansen et al., 2017; Moen et al., 2017; Frydendal and Thing, 2019).

Although most students enjoy PE lessons, student's experiences of stress might hinder the realization of PE engagement as a learning goal for *all* students. Blankenship (2007) argues that if students frequently experience negative stress in PE, this can reduce their enjoyment of physical activity and destroy the individual's desire to be a lifelong mover. Stressful experiences in PE might pose a risk for the increasing number of students that suffer from anxiety and depression symptoms. There is an increasing concern about school-related stress, especially among girls (Sletten and Bakken, 2016; Eriksen et al., 2017; Lillejord et al., 2017; Bakken, 2019; Sund et al., 2019). Røset et al. (2019) explored young people's perceptions and experiences of PE and their possible consequences for their mental health. Although students' experience of stressors during PE lessons has recently been given more attention (Tudor et al., 2018), research in this field is still limited. Young people's engagement in after-school physical activities seems heavily influenced by past PE experiences (Cardinal et al., 2013; Jaakkola et al., 2017). Consequently, it is important to identify contextual factors in PE that may negatively influence students' motivation, enjoyment, and participation. The aim of this case study was to identify potential perceived stressors among 9th-graders in a Norwegian PE context.

Stress is studied in various fields and can be conceptualized in several different ways. According to Lazarus and Folkman's (1984) transactional stress theory, it is the complex transaction between the individual and the environment that causes stress. The transactional stress theory focuses on coping processes that directly modify stressors and reduce emotional distress arising from negative individual and environmental transactions (Lazarus and Folkman, 1984).

A stressor, or a source of stress, was defined by Selye (1976) as “that which produces stress.” The assessment of whether the relationship between a person and the environment is stressful depends on the person's cognitive appraisal (Lazarus and Folkman, 1984). Cognitive appraisal is defined as “the process of categorizing an encounter and its various facets with respect to its significance for well-being” (Lazarus and Folkman, 1984). According to Lazarus (1999), people are constantly evaluating their relationship with the environment with respect to their implications for their well-being. Appraisals are strongly influenced by personality variables, meaning that two individuals can construe their situations quite similarly yet have very different emotional reactions because of the difference in their appraisal of the adaptational significance of a situation (Smith and Lazarus, 1990). What may feel threatening and be stressful for some is not for others (Lazarus and Folkman, 1984) because of past experiences and differences in available repertoires (Antonovsky, 1979). Psychological stress is defined as a particular relationship between a person and the environment appraised by the person as taxing or exceeding his or her resources and endangering his or her well-being (Lazarus and Folkman, 1984). The variation in students' appraisal in the same environmental context, like in PE, can be explained by differences in their agendas, consisting of their values, goals, and beliefs, and the complex nature of the external environmental contexts, such as demands and resources (Lazarus, 1991).

According to Lazarus and Folkman (1984), “daily hassles” are less dramatic stressful experiences that arise from our roles in life and seen as little things that can irritate and distress people. They argue that, although daily hassles are far less dramatic than major changes in life, they may be important for adaption and health (Lazarus and Folkman, 1984). Due to their cumulative and proximal nature, daily stressors have a significant impact on academic results in school (Tudor et al., 2018). Secondary school–based research on stressors has associated everyday academic stressors with general educational contexts, academic achievement, well-being, and negative attitudes toward school (Sletten and Bakken, 2016; Lillejord et al., 2017; Tudor et al., 2018). Tudor et al. (2018) argue that earlier findings regarding experiences with daily stressors affecting school results are unique to the experience in the classroom and therefore cannot be transferred to the PE domain. According to Redelius and Larsson (2010), students are particularly exposed and vulnerable in PE. Few studies have directly examined the concept of stressors associated with experiences in PE lessons from the student's perspective in lower secondary school (Blankenship, 2007; Elliott and Hoyle, 2014; Tudor, 2018; Tudor et al., 2018). A systematic review of stress among PE teachers found that to some extent, they experience more stress and burnout than other teachers (Von Haaren-Mack et al., 2019).

Recent studies (Tudor, 2018; Tudor et al., 2018) found stressors unique to the PE context, linked to the social, physical, organizational, and performance environment. Identified stressors like interpersonal relationships between peers, visual performance, and body exposure are consistent with earlier research (Elliott and Hoyle, 2014; O'Connor and Graber, 2014; Lyngstad et al., 2016; Wiltshire et al., 2017; Kerner et al., 2018). According to Hills (2007), PE represents a dynamic social space where students experience and interpret physicality in contexts that accentuate peer relationships and privilege particular forms of embodiment. Tudor et al. (2018) identified potentially frustrating environmental requirements that may affect participation. Despite this insight, we are far from fully understanding students' experience of stress and the causes of stress in PE. This knowledge gap is problematic given the potential negative and positive outcomes of students' adversity-related experiences and stress. As early adolescence is a developmental period associated with decreased engagement and participation in PE, it is important to identify the environmental stressors associated with increased disengagement.

A systematic review of the causes of school stress commissioned by Norway's Ministry of Education and Research (KD) highlighted the need for more qualitative studies using multiple data sources to examine the causes of stress at school from the student's perspective, especially regarding gender differences (Lillejord et al., 2017). Additionally, Tudor et al. (2018) suggested that future research may benefit from complementing focus group and interview data with observations of PE lessons.

### The PE Context in Norway

The primary purpose of PE as a general study subject in the Norwegian curriculum is to inspire a physical active lifestyle, lifelong joy of movement, and mastery according to each student's own skills and ability levels. Students should experience joy, thrill, and inspiration by participating in various activities with others (Norwegian Directorate for Education Training, 2015). PE is a compulsory subject in Norwegian schools and the third most taught in terms of teaching hours in grades 1–10 (Norwegian Directorate for Education Training, 2019). The lessons are coeducational [(The Education Act, 1998) §8.2]. However, in some cases, some teachers still practice gender-segregated teaching on the grounds that boys dominate in the subject, and regarding gender and religion in swimming lessons (Klomsten, 2013; Walseth et al., 2017). The main subject areas in lower secondary school (8th−10th grades) are sports activities, outdoor life (*friluftsliv*), exercise, and lifestyle. Grades represent the competence attained according to the curriculum description of each subject and the student's effort (Norwegian Directorate for Education Training, 2015).

### Aim

Through our review we have identified that much of the relevant research has been conducted outside Scandinavia and our study aimed to address this limitation in the current state of knowledge. Thus, this qualitative case study was aimed to take an emic approach to understand the informants' perceptions of contextual stressors and thus broaden the perspective of perceived stressors in PE for 9th-grade students in lower secondary school in Norway.

The research question was the following: *What, if any, are the contextual stressors perceived in PE lessons?* Using multiple data sources, we aimed to further examine this overarching question by addressing the following sub-questions:

- *What do students perceive as contextual stressors in PE lessons?*
- *What do teachers perceive as students' contextual stressors in PE lessons?*
- *What contextual stressors are observed in PE lessons?*
- *Are there any gender-specific differences regarding perceived contextual stressors in PE lessons?*

These research questions require that our ontological and epistemological positioning in our study are coherent with our methodology, and our case study is therefore situated in a social constructivist paradigm (Stake, 1995; Krumsvik, 2019).

This study was mainly concerned with exploring specific environmental stressors in PE that can potentially be negatively appraised.

## Methods

### Case Study, Participants, and Setting

This study relies on Stake's (1995) definition of qualitative case study research as “naturalistic, holistic, ethnographic, phenomenological, and biographic research methods” and “a palette of methods” (Stake, 2006, pp. xi–xii). The case framework we have applied can be described as an intrinsic case (Stake, 2006), where the intention is an emic and etic understanding particular of a single case in PE at one school, based on multiple data collection and “analytical eclecticism” (Thomas, 2011).

**Figure 1:**
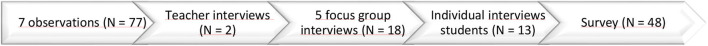
The cumulative research process.

This was a qualitative case study on 9th-graders' perceptions of stressors in PE lessons in one public secondary school in Norway (*N* = 77). It included observations from seven PE lessons (1 lesson = 60 min), interviews and informal conversations with two male PE teachers, five focus group interviews with 18 students, 13 individual interviews with students, and a supplementary self-reporting survey (Yin, 2009) based on prior preliminary research findings from the abovementioned methods (Figure 1). The self-reporting survey applied is a minor part of the study and based on a cumulative analyze process of qualitative data, which complement our understanding of the qualitative data material.

The strengths of this design include the ability to investigate the phenomenon in natural settings and the use of a variety of research methods to obtain rich descriptions and deep insights, enabling us to understand the students' engagement in practices and interactions in the PE context (Merriam and Tisdell, 2016). Triangulation of qualitative methods allows identification of possibly overlooked stressors, nuances in theory, and an emphasis on the importance of context.

### Ethics

Saunders's et al. (2016) model of stage-specific ethical issues and Tangen's (2014) ethical matrices contributed to our reflection on ethical issues and the interplay between ethics and internal and external quality in every stage of this study. The study was approved by the Norwegian Center for Research Data (NSD). The school's principal gave permission to conduct the project at the school before contacting the PE teachers. The students' legal guardians and their PE teachers and their assistants all gave their written informed consent before their participation in the study. The participants were informed that they were free to withdraw from the research at any time and for no reason, in which case, their recorded interviews would be deleted. To improve the reliability and comprehensiveness of the data extracts translated from Norwegian, grammar and spelling errors have been corrected. Quotations are referenced by pseudonyms to protect the participants' anonymity.

### Pilot Interviews

A pilot study (Åsebø and Innselset, 2017) was conducted in the school year 2016–2017, including 17 semi-structured interviews with PE teachers from 8 secondary schools in Norway. For the development of three different interview guides, three additional individual interviews were conducted, with two 14-year-old girls and a 39-year-old female PE teacher, and one focus group interview with three students, a 14-year-old boy, a 15-year-old girl, and a 16-year-old girl. A co-moderator was present during the focus group interviews, where topics related to stressors and unpleasant and negative experiences in PE were discussed. Interviews and subsequent discussions were not recorded, but both authors took notes diligently. Consequently, some questions and formulations were removed or revised, and new questions were added to target the research question. The process offered insight into how to approach this age group, how to respond to the students' responses, how to probe (Merriam and Tisdell, 2016), and how to create a safer conversation atmosphere for the participants.

### Observation

At this school, all three 9th-grade classes were merged and divided into two large groups. The first group started with PE, and the other with another subject. The groups were then divided into two smaller groups. Each PE teacher instructed a group of 17–20 students (mixed from all three classes) per lesson. Additionally, each class had one lesson per week separately.

Descriptive field notes were taken during seven PE lessons (*N* = 77), each lasting ~60 min (Supplementary Table 1). The lesson content varied between dance, volleyball, swimming, handball, and outdoor activities. To ensure the collection of detailed information about the context, we utilized additional field notes from informal conversations with PE teachers and students, supplementing documents (timetable, half-year plan), and pictures from different arenas.

The descriptive field notes were inspired by Merriam and Tisdell's (2016) checklist of elements important for observation (1) the physical settings, (2) the participants, (3) activities and interactions, (4) conversation, (5) subtle factors, and (6) the researchers own behavior. Naturalistic observations (Hastie and Hay, 2012) were focused on interactions with fellow students and PE teachers, the way in which the teacher organized and facilitated learning, students' participation in the activities, verbal and non-verbal communication, and body language.

Participant observations were made by the first author to understand the specific context, to triangulate and enhance the study's trustworthiness (Patton, 2015; Merriam and Tisdell, 2016), and to describe specific incidents and behaviors relevant as reference points for subsequent interviews (Merriam and Tisdell, 2016). Combining observations with interviews, so-called “anchored interviewing,” enables the researcher to ask students how they experienced different situations and what they were thinking during the PE lessons (Merriam and Tisdell, 2016).

Observational findings were related to stressors identified through the observer's interpretations of the students' body language, their statements, their interpretation of different assignments, and their movements in the room, as well as the teacher's and fellow students' reactions to their conduct in relation to the context. Examples of body language noted were looking uncomfortable, tense behavior, crossed arms, fidgeting with the fingers, embarrassment (blushing), looking down, wan eyes, movements in the environment (pulling out to the side, standing in the rear of the room, standing behind others), small movements, not completing movements, passivity, lack of effort, increased effort, eager gaze, concentration, looking around to see if others are watching, feeling exasperated, bothered, worried, uneasy, troubled, anxious, upset, disturbed, nervous, irritated, or agitated, withdrawal, and silence.

### Interview Guides

The development of the interview guides was a cumulative process (Aase and Fossåskaret, 2014) based on knowledge from past research theory, previous teaching experiences, piloting, and field notes. Semi-structured interviews allowed for a certain degree of standardization and at the same time flexibility, giving students the freedom to elaborate on questions that they felt were important to their subjective experiences (Sparkes and Smith, 2014). The interview guide was informed by Lazarus and Folkman's (1984) TST to ensure that core areas of interest were covered by focusing on stressors.

Theoretical concepts were operationalized into more mundane questions to make it easier for students to understand what we were asking for. We asked questions like; I am interested in listening to your experiences with PE. Can you tell me how you experience PE? What do you like/dislike about the subject? What is it that makes you feel good/not so good in PE? Can you tell me about any negative experiences in PE for you? What do you mean by stress and being stressed? I want to know about your experiences with stress in PE. Think about what happens in the PE lessons and in the locker-room before and after the lessons. If you get stressed, what factors can trigger stress in PE for you? What can possibly make you feel stressed in PE?

Using statements and probing (Merriam and Tisdell, 2016) made it easier for them to reflect up on their experiences in the subject and giving us the opportunity to explore the students recall of perceived stressors in the memorable past from PE lessons, students coping- and self-protective strategies employed. At the end of each interview, interviewees got the opportunity to speak about stress and unpleasant/negative experiences in PE, which they believed we had not talked about. Most felt they had said enough, while some elaborated on some elements a little more.

### Interviews

During spring 2019, the first author interviewed 13 students (7 girls and 6 boys) from three different classes in 9th grade and their 2 male PE teachers (Supplementary Tables 2, 3) Gaining access to the students' voices about experiences, perceptions, attitudes, and explanations of their social worlds in PE and generating rich and contextualized data are strengths of our interview methods. The opportunity to probe and follow up on both anticipated and unexpected insights gives the interviews more depth and richness (Merriam and Tisdell, 2016). Individual interviews were conducted in a familiar room at the school, so that the interviewees would feel comfortable enough to share their perspectives and feelings.

### Focus Groups

Listening to students' discussions may provide greater awareness of their perspectives on what can cause stress in PE. The selection of students was based both on a purposive sample, based on their qualifications according to the research question, and on an accessibility sample, based on giving written consent to participate in the study (Patton, 2015). In collaboration with their PE teacher, the groups were formed to reflect student diversity (maximum variation) and were thus based on initial observations. Other criteria were related to the students' ability to feel confident in the group to speak freely, the representation of both genders, and the inclusion of students who were both active and inactive in their leisure time, students with different grades, and students who exhibited little participation in the PE lessons. Eighteen students (ten boys and eight girls) were interviewed in five focus groups (some mixed and some gender-specific based on the abovementioned criteria), each consisting of three to four students (Supplementary Table 4). All focus group interviews were conducted with the second author as a co-moderator present after the observations and the teacher interviews. A key advantage of focus group data collection is that it allows access to social interactions and the way in which meaning is “negotiated” in context, which means that the participants' accounts need to be considered in context (Braun et al., 2016). The moderator's challenge is to create a benevolent and open atmosphere where the participants can express personal and contradictory views.

The focus group interviews were semi-structured, based on open-ended questions and statements to discuss. To stimulate interaction between the students, discussions were generally allowed to flow in the direction of their own answers. Efforts were made to contain students who tended to dominate discussions. At the same time, shy and reticent students were encouraged to contribute to the discussions, for instance by direct questioning and giving them the opportunity to participate by looking and nodding at them but respecting their wish to remain silent.

### Data Analysis

With each interviewee's permission, a Sony digital voice recorder (ICD-PX370) was used to record individual and focus group interviews, and notes were taken. For backup, we used two recorders during the focus group interviews. Experiences and first analysis from each interview were recorded in a digital diary immediately upon completion of each interview. All interview recordings were verbatim transcribed shortly after completion. Average duration of teacher interviews were 47 min, student interviews 29 min, and focus group interviews 46 min. Consisting of all together 387 pages of transcriptions. The transcriptions were then imported into the NVivo12 Pro qualitative analysis software for technical support and analyzed according to the principles of reflexive thematic analysis (Braun and Clarke, 2006; Braun et al., 2019). Braun and Clarke's (2006) six-stage process is flexible and can provide a rich and complex understanding. The first stage is familiarization with the generated data by listening to the recordings, transcribing interviews verbatim, repeatedly reading notes and transcripts, and looking at the data analytically. This is followed by coding and developing a map of themes and codes. The themes and codes were refined through an iterative process of reading, writing, and analyzing, keeping close to the participants' statements in the preparation of the theme structure. The themes were identified both at a semantic level as communicated directly by informants and at a deeper, more implicit, latent level (Braun and Clarke, 2006). This process is closest to what is theoretically referred to as abduction (Alvesson and Sköldberg, 2018). This abductive approach allowed us to be flexible, and using pre-identified themes allowed both using previous literature and remaining sensitive to new knowledge that could be constructed. In assessing the quality of this process, Braun and Clarke's (2006) 15-point “checklist” for a good thematic analysis was helpful. The theoretical assumption underpinning this analysis was social constructionist epistemology, which views meaning as the product of social processes and interactions (Burr, 2015). The qualitative orientation and use of the reflexive thematic approach emphasize the active role of the researcher in interpreting data and the knowledge production process. Where meaning is contextual, realities are multiple, and the researcher is seen as a resource (Braun et al., 2019).

In the first phase of the analysis, four overarching themes emerged as coherent through the four different methodical approaches. Next, the first and the second author agreed on every single quote into coding to subthemes and the categorization into main themes and overarching themes (Table 1). The coding and categorization were agreed upon taking into account all transcriptions.

**Table 1:** Example of the analysis process.

**Raw data from individual interviews with students**	**Subtheme**	**Main theme**	**Overarching theme**
Jon: *Well, if they have something they don't like at all, then they'll probably be a little stressed. Something that they are not good at and such*.	Personal preferences	Lesson content	Teaching environment
Sam: *[...] They [the teachers] can try to divide or create different stations with things and stuff, so whoever wants it, they can choose what they want to do, so they don't have to choose something they might not get and stuff like that. Then there will probably be a little less pressure because they know they will manage it*.			
Sarah: *Yes, I can get stressed when I'm very excited about winning in something; then maybe I get nervous and I start to shake a little, and then maybe I get a little tired before I start the competition [...]*.	Competition		
*Elsa: No, but I get stressed out if I see it's possible to lose*.			
James: *[...] If you are going to win, then you are usually a little stressed because you think, “What If I lose?” or “What if I can't do it?”*			

Merriam and Tisdell's (2016) strategies to promote validity and reliability were used to enhance the trustworthiness of this case study through the entire process. The first strategy was using multiple investigators and various data collection methods to confirm and reinforce important findings. The cumulative design and process in which the various methodological approaches build on each other and incorporates knowledge and situations from the observations into the interviews with the students in order to probe and ask follow-up questions related to observations from PE lessons. Observations revealed amongst others that some students seemed affected when working together with peers who were better at performing the given activity than themselves. “Anchoring interviewing” made it possible for us to follow up such observations for example by asking; *How do you experience working with others that you feel is better than you? For example, in PE when you were making a dance in small groups?* Secondly, discussing tentative interpretations from observations with the teachers in informal conversations and using tentative findings and interpretations in the self-reporting survey with students. The third strategy was adequate engagement in generating and collecting data, by being in the field in the period between 13.03.19 and 06.05.19 to obtain a satisfactory “amount” of data. Fourth, continuously reflecting upon one's ontological and epistemological position as researchers, as well as accounting for conditions that we believe may have influenced our interpretations of findings. The first author was mainly responsible for generating data by conducting and analyzing observations, focus group interviews, individual interviews and the supplementary survey. The first author is a 40-year-old female PhD candidate with an educational background in PE and sports pedagogy, with 15 years of prior teaching experience in PE and PE teacher Education (PETE). The second author, a 50 -year-old female associate professor, with 20 years of teaching experience was co-moderator during the focus group interviews and analyzes. The third author, a 53-year-old male professor was responsible for conducting, analyzing the supplementary self-reporting survey and the overall research design. The fifth strategy was peer reviewing by discussing the research process, strategies, tentative findings and interpretations with fellow researchers and colleagues. Making an audit trail to make a detailed account of methods, procedures, and important decisions within the study was our sixth strategy. The seventh strategy was to provide thick and rich descriptions to contextualize the study such that make it possible for readers to determine the extent to which their situations and findings match the research context. The last strategy was providing maximum variation and diversity in the sample selection. Based on observation and according to the PE teachers, students participating in focus group interviews and individual interviews represented a diverse sample in terms of fondness for PE, PE grades, gender, activity experiences, level, and skills.

### Self-Reporting Survey

The purpose of the survey was to function as a supplementary data source and minimize the most common validity threats: researcher bias and reactivity (Maxwell, 2009) in field work. More specifically, the survey was conducted in the final stage of a cumulative data analysis process where we wanted to identify and check for diversity vs. uniformity in our data material, in order to avoid eventually biases overlooked earlier in the data analysis process. Such check for supporting evidence as well as negative evidence aims to increase the internal generalizability (Maxwell, 2010) between participants and methods as a whole in the case, in order to avoid the claim of cherrypicked data for only supporting interpretations. It was conducted “live” (spring 2019), by the first and the third author, with all students gathered in a lecture hall (*N* = 48; 52% female, 48% male; response rate: 95%) using a student response system (SRS; TurningPoint) during a school hour (45 min). The survey consisted of three sections: (1) demographic information, (2) questions regarding PE, and (3) statements and questions regarding self-perceived stress in PE. The survey was designed to examine whether the preliminary findings from other qualitative data sources in the case study (Figure 1) corresponded to the rest of the 9th-grade students at the specific school. Such triangulation is essential for gaining a more thorough understanding of the research questions, as well as to protect the data, and ultimately the conclusions, from validity threats. Quantitative data were analyzed using descriptive statistics (frequencies, means, and standard deviations). An independent sample *t*-test was used for group differences between girls and boys. All analyses were performed using IBM SPSS Statistics 21. Missing values and incomplete inputs were removed before the analysis in order to maintain a complete respondent dataset (Tolmie et al., 2011).

### Naturalistic Generalization

Since this qualitative case study don't aspire to carry out any statistical generalization and rather aims to understand and explain a concrete reality in the specific context of the study, we position this qualitative case study to naturalistic generalization (Stake, 2010; Krumsvik, 2019).

## Results

Through this case study, both the prevalence of perceived stress in PE and the many different facets of stressors associated with it became visible. In the individual interviews, three out of 13 students stated that they felt more stressed in PE than in other subjects. The findings show that, although some students experience considerable stress, most students experience less stress in PE compared to other subjects. According to one of the teachers, PE seemed to relieve stress in some students:

I really feel that in PE, the students are least stressed. Many students are stressed about grades of course, especially in secondary school, and they experience the grade pressure and that kind of thing and feel that they must perform and perform. But in my PE classes, I feel that many students unwind and do not feel that they must perform. (Kane)

The qualitative analysis generated four overarching themes of environmental demands that could potentially be appraised as harmful by the participants: (1) teaching environment, (2) physical environment, (3) social environment, and (4) personal factors. This wide span of themes illustrates the complexity of stress perception in PE.

The subthemes, presented in Tables 2, 3, form the substance of the analysis, with relevant extracts from the interview transcripts textualizing students' and teachers' voices and demonstrating the interpretive adequacy of the analysis. The tables are syntheses of all the findings from the qualitative methods, in order to facilitate a comprehensive overview. We'll elaborate on some key findings below.

**Table 2:** Perceived contextual stressors in PE lessons.

**Overarching theme**	**Observation**		**Teacher interviews**		**Focus group interviews**		**Individual interviews**	
	**Main theme**	**Subtheme**	**Main theme**	**Subtheme**	**Main theme**	**Subtheme**	**Main theme**	**Subtheme**
Teaching environment	Lesson content	Activities with significant level differences	Lesson content	Activities with significant level differences	Lesson content	Activity	Lesson content	Activity
						New activity		Competition
		Activities		Type of activity		Competition		Personal preferences
		Competition		Swimming		Lack of knowledge about training		
		Performance		Hard sessions		Afraid of getting hurt/injured		
		Involuntary role				Uncomfortable activity		
	Methods and organization	Lack of information	Methods and organizatn	Lack of information	Methods and organization	Lack of information	Methods and organization	Lack of information
		Visibility		Visibility		Visibility		Visibility
		Time pressure		Perceived progress		Time pressure		Time pressure
		Difference in skills within group		Dividing into teams/groups/pairs		Lack of variation		
						Personal equipment		
		Being watched				Dividing into teams/groups		
		At the beginning of the lesson				Lack of adaptive education		
		Constantly being in the same group						
		Teacher's attention						
		Too difficult						
		Too easy						
		Lack of structure						
		Skillful students as co-teachers						
		Queue						
		Long distances						
	Assessment	Demonstration of skills	Assessment	Teacher's grade pressure	Assessment	Grade pressure	Assessment	Grades
				Physical tests		Testing		Testing
				Skills becoming evident in competitive situations		Not participating		Not participating
						Demonstration of skills		Teacher's expectations
						Teacher's expectations		Parents' expectation
						Assessment criteria		Assessment criteria
	Teacher	Teacher's competence	Teacher	Teacher's expectations	Teacher	Teacher's competence	Teacher	Not being specific
		Lack of instruction		Teacher's comments		Teacher's gaze		
		Focus on performance		Student–teacher relationship		Authoritative teacher		
		Teacher nagging						
Physical environment	Equipment	Afraid of getting hurt/injured	Equipment	Poor equipment	Equipment	Afraid of being injured by the equipment		
				Afraid of getting injured/hurt				
	Facilities	Limited space	Indoor facilities	Limited space	Facilities	Several students in the locker room		
		Several students in the locker room	Outdoor facilities	Others watching		Being in a new space		
						Space		
					Weather	Rainy weather/snow		
					Class size	Big class		
Social environment	Social comparisons	If others make it and I don't.	Social comparisons	Afraid of ruining it for others	Social comparisons	If others make it and I don't.	Social comparisos	Rivalry
		Lagging behind		Personal exercise equipment		Lagging behind		Performance climate
		Afraid of making mistakes		PE grade giving status		Afraid of making mistakes		If others make it and I don't.
		Afraid of ruining it for others		Body pressure		Afraid of ruining it for others		PE grade giving status
		Performance climate		Body exposure		Grades		
		Body size				Puberty		
						Body image		
						Rivalry		
	Expectations	Cheating	Expectations	Parents' grade expectations	Expectations	Angry fellow students	Expectations	Angry fellow students
		Game expectations		Preserving one's reputation				Not living up to one's own expectations
		Others ruining the game						Others depending on you
		Preserving one's reputation						Feeling like a burden
	Friends	Being with someone you don't know	Friends	Class environment not feeling safe	Friends	Class environment not feeling safe	Friends	Classroom environment doesn't feel safe
		Body contact				Being with someone you don't know		Being dependent on others
		Tension between genders						
	Comments	Students not participating			Comments	Body shaming	Comments	Affecting one's grade
		Disagreements				Fellow students nagging		Others thinking they are better than you
		Fellow students nagging				It depends on who one gets a comment from.		Scary comments
						Others talking behind one's back		
	Exclusion	Low skill			Exclusion	Low skills	Exclusion	Selfish boys
	Skillful students	Gloating	Skillful students	Inability to see others	Gaze	Body pressure	Gaze	Fellow students staring
		Criticizing		Negative comments		Embarrassment in front of others		“Bitch Blink”
		Giving the premise of the lesson		Negative body language		Afraid of being photographed in the locker room		
				Desire to win				
	Body language	Disappointment					Body language	Others laughing and whispering
	Collaboration	Poor collaboration	Social media	Comments				
			Mental health	Diverse pressure				
Personal factors	Self-efficacy	Lack of mastery	Self-efficacy	Lack of mastery	Self-efficacy	Lack of mastery	Self-efficacy	Lack of mastery
		Past experiences		Past experiences		Past experiences		Afraid
		Losing in competitions		Afraid of failing		Afraid		
		Afraid						
	Body dissatisfaction	Exhaustion	Body dissatisfaction	Not accepting the way one looks	Body dissatisfaction	Not fit	Control	Losing things
						Exhaustion		Lack of time
						Body exposure		Lack of control
						Not accepting the way one looks		
			Mindset	Negative thoughts	Mindset	Negative thoughts	Mindset	Negative thoughts
			Perceived competence	Exhaustion	Perceived competence	Feeling like a failure		
						Not feeling good enough		

**Table 3:** Synthesized findings from all qualitative methods.

**Teaching environment**	**Physical environment**	**Social environment**	**Person factors**
**Lesson content** Activities Type of activity New activity Uncomfortable activity Personal preferences Competition Swimming Performance Involuntary role Hard sessions Lack of knowledge about training Afraid of getting hurt/injured **Methods and organization** Lack of information Visibility Time pressure Difference in skills within group Being watched At the beginning of the lesson Constantly being in the same group Teacher's attention Too difficult Too easy Lack of structure Skillful students as co-teachers Queue Long distances Perceived progress Dividing into teams/groups/pairs Lack of variation Personal equipment Lack of adaptive education **Assessment** Grade pressure Testing Not participating Demonstration of skills Teacher's expectations Assessment criteria Parents' expectation Teacher's grade pressure Physical tests Skills becoming evident in competitive situations **Teacher** Teacher's competence Lack of instruction Focus on performance Teacher nagging Teacher's gaze Authoritative teacher Not being specific Teacher's expectations Teacher's comments Student–teacher relationship	**Equipment** Afraid of getting hurt/injured Afraid of being injured by the equipment Poor equipment **Facilities** Limited space (inside) Others watching (outside) Several students in the locker room Space Being in a new space **Weather** Rainy weather/snow **Class size** Big class	**Comments** Students not participating Disagreements Fellow students nagging Body shaming It depends on who one gets a comment from. Others talking behind one's back Affecting one's grade Others thinking they are better than you Scary comments **Social comparisons** If others make it and I don't. Lagging behind Afraid of making mistakes Afraid of ruining it for others Grades Puberty Body image Rivalry Performance climate PE grade giving status Personal exercise equipment Body pressure Body exposure Body size **Expectations** Cheating Game expectations Others ruining the game Preserving one's reputation Parents' grade expectations Angry fellow students Not living up to one's own expectations Others depending on you Feel like a burden **Skillful students** Gloating Criticizing Giving the premise of the lesson Inability to see others Negative comments Negative body language Desire to win **Friends** Being with someone you don't know Body contact Tension between genders Classroom environment not feeling safe Being dependent on others **Gaze** Body pressure Embarrassment in front of others Afraid of being photographed in the locker room Fellow students staring “Bitch Blink” **Body language** Disappointment Negative body language Others laughing and whispering **Exclusion** Low skills Boys **Collaboration** Poor collaboration **Social media** Comments **Mental health** Diverse pressure	**Self-efficacy:** Lack of mastery Past experiences Losing competitions Afraid Afraid to fail **Body dissatisfaction:** Not fit Exhaustion Body exposure When you don't accept the way you look **Control:** Losing things Lack of time Lack of control **Mindset:** Negative thoughts **Perceived competence:** Feeling like a failure When one doesn't feel good enough Getting exhausted

### Teaching Environment

The first theme related to the teaching environment and how the teacher facilitates learning. The PE teachers educational and didactical choices, lesson content, how the teachers organize the lessons, teaching methods, teaching principles and practice. Within the main theme lesson content, we identified a range of different stressors shown like subthemes in Table 2. Summarizing the different methodological approaches, we found that lesson content and what type of activity they had in PE was of great importance of whether the students were stressed or not, swimming was one of those activities.

I am not so fond of swimming, so I get a little stressed by swimming, [...], I am average, but I think it sticks from primary school, because then I was a bit stressed out in the pool because of technique and not being as fast as the others. (Sue, Ind. Int.).

Students personal activity preferences, new activities and past experiences with different types of activities seemed to have great significance for the students' experiences:

I really like ball games and stuff, I really like almost everything. I just don't like volleyball very well, because I get really hurt in my hands. Because I am not very good at the volleyball technique. Otherwise I like PE very well. (Jaxon, FG 4).

Probably because they've hurt themselves before. I know of someone who tries to avoid, or who often does not participate in PE, because they often hurt themselves. Or I know about one person. (Andrew, FG 1).

Within the main theme methods and organization most stressors were identified during observations. Subthemes common to all methodological approaches were; lack of information, visibility and time pressure. Sue a skillful handball player was stressed by lack of information:

I get stressed, for example yesterday when we had handball, […] when the other team didn't get good enough explanation. […]. I got really stressed when I know in a way how it should be on a handball court, and I had no idea what to do and I did not get to show my skills when it's not proper. (Sue, Ind. Int.).

Observations showed that depending on how the teacher organized the lessons, type of activities, what equipment they had and how they used it, how they used the facilities and how they divided the students into groups, visibility seemed to be of importance. Students feeling visible and exposed in different settings. PE teacher Kane explained:

They think it' [PE] is uncomfortable because the others can see it. For example, I had a student who did not want to have PE at all, because then he felt that everyone was watching him. Not only in his class, but everyone around in a way and felt it was uncomfortable.

How to divide into groups, difference in skills within the groups and how long the groups cooperate were common stressors. According to the two PE teachers, they were very conscious that they should divide into groups and not the students themselves, to avoid anyone feeling left out.

We found several stressors related to the facilitation of teaching and what didactic considerations were made within the lesson. Observations also revealed that situations like at the beginning of the lessons, especially before swimming, when being watched at from the sideline and when queuing seemed stressful for some students. This was also expressed both in focus group interviews and during individual interviews.

I bet that even if we are good in PE, no one ever wants to stand first in line. There is always someone pushing people in front of us in some way. And then they must start right, so there are very few who will stand first in line and when they don't quite right. And it's all about being sure of the exercises. (Daisy, FG 3).

### The Physical Environment

The Physical environment related to how the facilities and frameworks factors at the school affect teaching and the student's experiences. Within the main theme equipment, students being afraid of getting hurt or injured by the equipment and poor equipment were identified as stressors for some students.

Ian: When we have gymnastics for example and then we should do different jumps on the trampoline and buck and stuff. Then there may be some people who don't really want to, because they are afraid to hurt themselves. But I haven't seen anyone who hasn't done it.
R: How do you think it was experienced by the individual?
Ian: That they are pretty scared and really don't want to do it, but then they just do it because everyone else does it.
Anna: I think they might get, I don't know if they'll get a kind of anxiety, but they get pretty scared and then they worry about the next lesson and when they hear “Oh we'll have gymnastics, we'll do it again,” so they like dread it and then they use all their energy to dread that thing. (FG4)

Lucy and Isabell discussed big size classes.

Lucy: Yes, in elementary/primary school we were just 11 in the PE class and then we didn't think about it, because we were very close. We didn't care what the rest did and stuff, but now we're about 30 students that like look at you.
R: So, big groups then (interrupted by Isabell)
Isabell: Yes
R: Does that matter?
Lucy: Yes (nodding consent), it's stress. (FG 2)

### Social Environment

The third theme related to the social environment in the class and how fellow students and teachers interact with each other. It's obvious that the presence and behavior of fellow students is of great importance for student's well-being in PE. From focus group interviews it became evident that students perceived social environment as the richest area of all stressors (Table 2). Within the main theme social comparison, were students compared themselves to each other in different ways and settings, were found in all the methodological approaches. Freddie in focus group five said; “If others make it and you're the only one who doesn't, then it can get pretty stressful.” Even two who could be defined as skillful students Mason and Daisy were afraid of ruining it for others:

Mason: Or you feel that you go together with someone who is very good, and you are not that good, you feel that you are ruining it for him.
Daisy: Yes.(FG 3)

Body exposure during swimming lessons was discussed by Daisy and Sophia during a focus group interview:

Daisy: Surely someone who is not entirely comfortable showing off their body, because one goes almost completely naked (laughs) with a little garment on.
Sophia: Yes, the swimsuit is pretty tight and…
Daisy: Yeah, it's kind of just a color, because it's so thin. So, you actually feel pretty naked.
Sophia: Yes. (FG3)

Expectations was also defined as a main theme:

[...] they are under pressure because they must perform those who are skillful too. Particularly those who do individual sports or who have a reputation for being athletic, they must keep up all the time to maintain that reputation or the “image.” (Kane, PE teacher).

The other PE teacher, Tim, spoke about parent's grade expectations:

Tim: [...], maybe parents' expectations play a role. When I have had complaints in PE I often feel that it is the parent who complains, because they expect the student to get a certain grade. Because the kid is skillful in sports, they think he should have top grades in the subject. I think it stresses the students that parents expect them to get a good grade and when they get a lower grade than they expected, it becomes a somewhat unfavorable situation simply.

Another aspect is not living up to one's own expectations:

Yes, if one is stressed then it may be because one feels that one may have to live up to some expectations in the subject and then things are not going as well as you expect and, or you are doing poorly. And then you want to, yes you feel a little bit stressed to do it maybe and that's it yes. (David, Ind. Int.).

Not feeling safe found in all tree interview approaches but identified as being with someone you don't know during observations:

Jaxon: If you have a class that is very like that, you have a very nice class. Then you really haven't anything to worry about in PE lessons.
R: What do you think about it (turns to Ian)?
Ian: No, if you know everybody, it's pretty safe. If you don't know anyone, you are more afraid of making mistakes and such. (FG 4).

The relevance of stressful comments was also discussed:

Evie: Yes, it's just the guys who comment on the girls and say, “My God you have to go all in and or” (laughs).
Isabell: “Serr” [seriously], “LOL.”
Evie: “My god this I could have done backwards, blindfolded” (laughs)
Isabell: The boys are a bit haughty/arrogant.
Evie: Yes, they are smug.
R: If I understand you correct, comments from the guys in class... (interrupted by Isabell)
Isabell: Or the girls
R: Or the girls, can make you feel stressed out in different situations?
Evie: Yes, mmm
(All the girls nodded in agreement). (FG 2)

Furthermore, others talking behind one's back, affecting one's grade, scary comments and others thinking they are better than you.

It is probably commentary or [...], if someone is trying to rise above others then it can be a little stressed out. (Sue, Ind. Int.).

Students spoke of exclusion because of low skills especially by boys, this was also found during observations, but not only by the boys. Observations showed that during a handball match one girl in particular were not being included in the game by not getting the ball from skillful students. A skillful girl was observed constantly throwing the ball to other skillful students instead of throwing the ball to a girl on her right, even though she was in a much better position. Skillful students appaired to be divided into two types according to the PE teacher Tim;

There are two different types of “able” students. You have the “able” students who are very self-absorbed who only work with themselves and want to win everything. They see competition in everything that happens. They are unable to see that there are some students who are “less able,” instruct and help them. In many contexts they have a negative impact on the rest of the class. But then again, we have many “able” students who are incredibly good at taking care of all students in the class, can work with everyone and help instruct as best they can. Adjust their level to suit the group, they help the others.

Skillful students' inability to see others and who were gloating, criticizing, giving the premise of the lesson, made negative comments, used negative body language and had a desire to win appeared as stressors both during the observations and teacher interviews.

Gaze as main theme was mainly spoken of by students both during focus group interviews and individual interviews as perceived stressors during PE. Fellow students staring, “Bitch Blink” when girls are giving each other dirty looks, body pressure, embarrassment in front of others and students afraid of being photographed in the locker room.

Body language as a main theme was observed as disappointment and spoken about in individual interviews as others laughing and whispering.

Other potential stressors were poor collaboration found during observations, comments in social media and the consequence for student's mental health due to diverse pressure.

### Personal Factors (Student-Related)

Finally, personal factors related to the student's assumptions, thoughts (how the students think, perceptions, attribution), past experiences, mastery and expectations of PE. Within the main theme self-efficacy identified through all four methodological approaches; lack of mastery was a stressor frequently mentioned. When asked what factors may trigger stress in PE during an individual interview, Pete answered:

It is probably if I am much worse off than the others, yes and when I don't master anything at all as I said and yes, that isn't so much fun [a little crying in his voice].

Both teachers and students mention the significance of past experiences and being afraid of failing. Loosing in competitions was also observed as a potential stressor.

Where one does not master. Where there is no self-confidence. Where you have negative experience from earlier. “Last time we had it, it went so and so.” Yes, it's a kind of fear that the same thing will happen again. (Tim, PE teacher).

Another main theme was body dissatisfaction identified during observations, teacher interviews but mainly discussed in focus group interviews. Consisting of subthemes like not accepting the way one looks, not being fit, body exposure and exhaustion:

When you know you're going to get very exhausted, then you can get very stressed, because when you know that “ok, now I'm going to get dead beat,” that I don't want to, but in one way to be a little stressed out. (Isabell, FG2).

Based on an overall impression, the students' mindset is of great significance. This, of course, was not obvious during the observations, but reflected through the other three approaches. The students' own negative thoughts seemed to be a significant stressor for some. Furthermore, perceived competence as a main theme consisted of exhaustion the feeling like a failure and not feeling good enough found within teacher interviews and focus group interviews.

Finally, lack of control as described by James during an individual interview:

When I'm stressed then I have no control. I like to at least have some control and then when I am stressed then I have no idea what is going on and so if I do not know what to do, then it is worse.

As shown above, several themes are interrelated and overlapping, demonstrating the nuances and complexity of students' experiences of potential and perceived contextual stressors in PE.

### Survey Results

**Figure 2:**
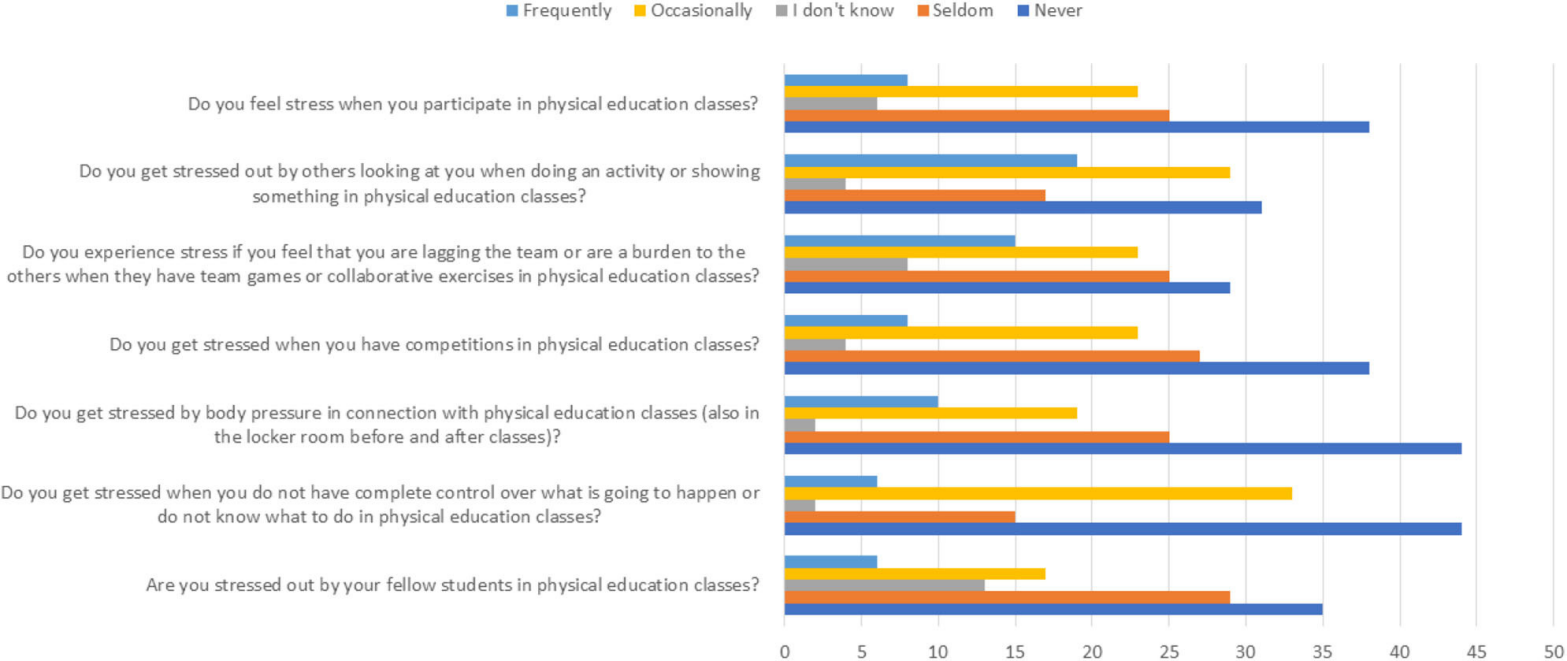
Students' self-reported data related to validation of the preliminary findings from the observations, focus groups, and interviews on an adjectival Likert scale, expressed in percentages (*N* = 48).

The self-reporting survey, based on prior preliminary research findings, helped us to determine whether our qualitative data were representative of the broader population (all 9th-graders at the school). Figure 2 presents (in frequencies) the PE survey results regarding self-perceived stress.

As between observations, focus groups and interviews, we found both convergence and inconsistency and contradictory findings (Mathison, 1988) regarding self-perceived stress from PE in the survey data, which especially indicates that some students are more vulnerable to contextual stressors than others. Table 4 shows gender differences expressed as means with standard deviations and *t*-test values.

**Table 4:** Contextual stressors by gender.

**Variable**	***N***	**M**	**SD**	***t***	***P***
Participate in PE				−2.63	0.012
Boys	23	1.87	1.25		
Girls	25	2.88	1.39		
Task difficulty				−4.33	>0.001
Boys	23	1.78	1.04		
Girls	25	3.32	1.38		
Being observed				−5.32	>0.001
Boys	23	1.87	1.14		
Girls	25	3.8	1.35		
Competitions				−2.77	0.008
Boys	23	1.83	1.23		
Girls	25	2.88	1.39		
Low self-efficacy				−4.01	>0.001
Boys	23	1.91	1.12		
Girls	25	3.4	1.41		
Cheering				−2.03	0.048
Boys	23	1.57	0.90		
Girls	25	2.20	1.22		
Trial and error is accepted in PE.				2.41	0.020
Boys	23	4.22	1.00		
Girls	25	3.52	1.00		
Other students				−2.81	0.007
Boys	23	1.78	1.13		
Girls	25	2.76	1.27		
Body image pressure				−3.07	0.004
Boys	23	1.65	1.19		
Girls	25	2.84	1.46		
Spectators				−2.54	0.015
Boys	22	1.45	0.86		
Girls	25	2.12	0.93		
Lack of control				−2.7	0.01
Boys	23	1.87	1.32		
Girls	25	2.96	1.46		

*M, mean; SD, standard deviation*.

Including all the significant findings, Table 4 shows that girls are the more vulnerable gender to contextual stressors, generally scoring higher on PE-related stress than boys. The analyses show that spectators, in addition to difficult tasks and low self-efficacy, seem particularly stressful for girls.

## Discussion

The overall purpose of this study was to identify and synthesize students' perceived stressors in different PE contexts that can potentially be negatively appraised. Giving 9th-grade students a voice and using multiple methodological approaches, we aimed to provide novel and deep insight that could be used to develop an understanding of the students' experience of stress while attending PE lessons in lower secondary school.

Our findings show that most students experience little or no stress in PE, some experience a little, depending on the situation, and a few of them experience considerable stress. In general, more than 35% of the students never experienced stress in PE, and an additional 25% seldom experienced stress. On the other end, at least 15% of the students frequently experienced stress in various PE situations—a tendency most evident among girls. These findings are consistent with previous research related to school stress (Sletten and Bakken, 2016; Eriksen et al., 2017; Lillejord et al., 2017; Bakken, 2019; Sund et al., 2019). The results are worrisome, as frequent experience of negative stress in PE can reduce students' enjoyment of physical activity and the desire to move (Blankenship, 2007) and negatively affect their academic results, and thus possibly their mental health (Røset et al., 2019).

According to Lazarus (1999), environmental demands and the conflicts that they can create with a person's inner goals and beliefs are among other obvious sources of psychological stress. He argues that the way in which a person copes with these demands, conflicts, and emotions arising from the struggle can influence the person's morale, social functioning, and physical well-being. Although the broad scope of stressors identified in this case study can potentially influence students in a negative manner, many of them could possibly be controlled or eliminated with the development of best PE practices.

### A Multitude of Stressors

Students experience a multitude of stressors during PE lessons depending on the context: the lesson content, the student's past experiences, how the teacher facilitates learning, who is involved, and how the students appraise the stressors. Within the different methodological approaches in this case study, most stressors coincide in the main themes organized under the four overarching themes: (1) teaching environment, (2) physical environment, (3) social environment, and (4) personal factors. There were mostly similarities but also some differences in what students perceive as stressors, what teachers perceive as students' contextual stressors, and what contextual stressors are observed in PE lessons. Subthemes include a multitude of stressors, which indicates that different students are influenced by different contextual stressors, and some are more vulnerable than others.

Within the main theme methods and organization, we identified more subthemes and situational details, such as potential stressful situations at the beginning of the lessons, long distances within the organized activity, when queuing, when skillful students were acting as co-teachers, the teacher's attention, and different levels of difficulty (too easy or too difficult). In all approaches, we identified some common stressors related to lack of information, visibility, being divided into teams or pairs, and time pressure. Consistent with Cardinal et al. (2013), group collaboration and team selection are challenging and potentially negatively associated with PE.

Moreover, the main themes of lesson content, assessment, and teacher were identified in all samples, which lends the findings coherence. Students seem to be very concerned about grade pressure and expectations. Both students' successes and shortcomings are more visible in PE than in other subjects (Redelius and Larsson, 2010). According to Säfvenbom et al. (2015), PE favors those already involved in physical activity, and especially those competing in sports. For some students, the teacher's lack of competence, variation, and organization skills hampers the experience of being in a supportive social climate. Additionally, when PE teachers are stressed, their stress may also influence the students' contextual experience of stress (Von Haaren-Mack et al., 2019). Relevant for the understanding of environmental stressors, Achievement goal theory (AGT) (Nicholls, 1989; Ames, 1992; Roberts and Treasure, 2012) distinguishes between a performance-oriented (ego-involving) climate, were individuals' ability and improvement are judged against fellow students' comparison and normative standards, mistakes or poor performance are somehow punished, and able students are consistently given praise and more attention. In contrast to a mastery-oriented (task-involving) climate, were the individuals' effort and improvement are recognized, every participant contribution is valued, and cooperation is fostered. The climate fostered by the PE teacher seemed to have a negative influence and was evident in certain activities and situations during observations and student interviews. In order to include all students in PE, a mastery-oriented climate might have greater potential (Nicholls, 1989; Ames, 1992; Ommundsen, 2004). Research employing AGT have shown that mastery-oriented climates are associated with many positive outcomes, whereas performance-oriented climates have been associated with more maladaptive outcomes (for a review see Roberts and Treasure, 2012). A recent meta analytic review reveals gender differences within certain sub areas of AGT (Lochbaum and Gottardy, 2015), but the current state of knowledge is still limited and thus we need more research within this area.

Stressors associated with the physical environmental are mainly related to equipment and facilities. The spatial experience appears to be challenging, especially in the locker room, where there is little room to hide, and when the activity takes place either in too small or too large an area depending on the group size. These findings are consistent with recent research (Johansen et al., 2017; Moen et al., 2017; Frydendal and Thing, 2019) showing that the presence of many students, especially when showering, are frequently mentioned by students as a stressor. In some situations, students felt that others were watching, for example when running outdoors, having too large space. Poor equipment, negative past experiences with similar equipment, and fear of getting hurt or uncomforted were perceived as stressful. Fear of getting hurt could diminish the eagerness to try new activities. Teachers point to poor equipment as an important stressor. Rainy weather and snow were also mentioned by students as perceived stressors.

Stressors arising from the social environment seemed to be most important. Students comparing themselves to others, being afraid of making mistakes, lagging behind others, ruining things for others, receiving negative comments, facing the anger of fellow students, and an unsafe class environment were some of the most frequent stressors found. Losing face in the eyes of fellow students and teachers may affect students' self-perception (Ommundsen, 2004). How the activity is perceived in relation to others is highly related to past experiences (Groves and Laws, 2000). Stressors such as comments, expectations, or exclusion might be the result of a performance- oriented climate favoring performance and social status.

Students have difficulties understanding the tacit messages and hidden meanings that are conveyed through their group relations and interactions (Munk and Agergaard, 2018). According to Nielsen and Thing (Nielsen and Thing, 2019), belonging to a group is a dynamic process. Students seemed to have a need for inclusion and a “we–I” balance were they constantly negotiated their belonging and concerning about how they present themselves. Issues like puberty and having a body in change, gaze, “Bitch Blink,” (when someone sends angry, critical and negative looks to others) and the influence of social media make the social environment even more complex. This supports previous research (Fisette, 2011, 2013) showing that many girls are concerned about being watched, observed, and evaluated based on physical appearance and skills by fellow students and teachers, which makes them feel uncomfortable and insecure. Girls being excluded and ignored by boys (Fisette, 2013) and the social and embodied dynamics amongst girls in PE (Hills, 2007).

In addition to environmental stressors, several stressors due to personal factors are also involved in PE lessons. Even though we haven't looked at the students' goal orientation (ego- or task-involved, Nicholls, 1989), it may be worth mentioning that previous research has shown that individuals with an ego- involvement has more anxiety concerns about performing, as opposed to task-involved individuals, because their competence and self-worth are not threatened (see Roberts, 2012 for a review). Lack of mastery, negative past experiences and fear of failure are some examples of student appraisals of stressors relevant for their self-efficacy beliefs. Students' perceptions of their own competence and performance are of central importance for the choice of activities and strategies, motivation methods, objectives, efforts, endurance, and performance levels (Skaalvik and Bong, 2003). Frequent negative experiences in PE might on an individual level hamper self-efficacy beliefs. Perceived self-efficacy is according to Bandura (1977, 1997) a person's confidence in their own abilities or believes in what one can do under different conditions, with the skills one possesses. Hence, people with similar skills or the same person may perform differently under different circumstances depending on their self-efficacy (Bandura, 1997). Bandura (1997) differentiated between efficacy expectations, the belief to successfully perform the behavior necessary to produce the outcome, whereas outcome expectations is the person's judgement that a given behavior will lead to specific outcomes. According to Bandura (1997) these two are differentiated, because the individual may believe that a particular course of action will give a certain outcome, but if the individual is in doubt of their own capability to perform or skills needed to succeed, it will affect the conduct. In this way, expectations of personal mastery will affect both initiation and perseverance in the coping behavior. The strength of the person's belief in their own abilities may affect weather they'll try to handle the given situations and influences the choice of behavioral strategies (Bandura, 1997). Bandura (1977, p. 194) explains that “People fear and tend to avoid threatening situations they believe exceed their coping skills, whereas they get involved in activities and behave assuredly when they judge themselves capable of handling situations that would otherwise be intimidating.”

Physical/motor self-esteem can be of great importance to young peoples' general self-esteem and psychological well-being (Haugen et al., 2014). Students feeling that they lack mastery do not enjoy these benefits in PE lessons. Body dissatisfaction, that is, not being fit enough or not accepting the way one looks, reflects a stressful mindset related to PE. Some students also have negative thoughts. These findings are consistent with Kerner et al. (2018), who found that students reporting greater body dissatisfaction also reported low perception of competence. Contrarily, students who perceived themselves as more physically competent in PE were more likely to report less body dissatisfaction. Kerner et al. (2019) found that students who felt more comfortable and satisfied with their physical appearance seemed to value and enjoy physical lessons more.

Although this case study included students from only three different classes, there are reasons to believe that perceived stressors in PE lessons are common across schools, and that there is much to learn in order to understand them and realize the importance of a safe and mastery-oriented social and teaching environment. Our findings show a multitude of realities and the complexity of students' experiences and perceived stressors in PE, corroborating Lazarus and Folkman's (1984) statement that what may feel threatening and stressful for some is not for others because of past experiences and available repertoires (Antonovsky, 1979).

Method triangulation seems to be a fruitful approach to understanding stressors in PE. While some themes emerge from all data collections, certain details appear in more richness in some approaches than others. In particular, the focus group interviews reveal details of social interactions and of the ways in which students relate to others. Students perceive stressors related to the social environment much more intensely than teachers do. This is an interesting finding, which supports the notion that relationships and the need to belong in a group are most important for understanding the motivational climate and enjoyment in PE (Jaakkola et al., 2017; Nielsen and Thing, 2019). Moreover, the relation between the difficulty perceiving students' experiences and the teachers' concern with broader themes is unclear. Teachers also seemed to be concerned about other themes such as social media and poor equipment. Observations were richer in detecting variation regarding the way that stressors are perceived as a consequence of methods and organization of the PE lessons. This could be explained by the experienced observer. While students' experiences are difficult to observe, the many observed situations served as a reference point for “anchored interviewing” (Merriam and Tisdell, 2016). The survey supports the main findings related to perceived stress in PE lessons, which are in line with previous research (Tudor, 2018; Tudor et al., 2018). Thus, this case study's approach provides rich, extended, nuanced, and differentiated insights into perceived contextual stressors in PE lessons.

Redelius and Larsson (2010) argue that the organization of PE is a key challenge to ensure that PE meets the needs of all students, and especially those not engaging in organized sports. If organization is not taken seriously, there is a risk that PE meets the expectations of students with an extensive experience in different sports at the expense of those with less experience and interest.

### Limitations, Strengths, and Future Directions

Reflexivity through this research process has revealed both the strengths and the limitations of this work. There are potential limitations to the students' comprehension of the language used when discussing such a complex subject. On the other hand, in this case piloting, the researchers' background, the time they spent in the field, and the use of multiple methodological approaches and triangulation are obvious strengths. Further research may benefit from a multiple case study design reflecting a wider range of PE contexts and teachers' and students' voices. Additionally, listening to a more diverse student group in terms of ethnic background would be of interest. Since cognitive appraisal of a given situation determines whether, how, and to what extent coping is appropriate (Lazarus and Folkman, 1984), an aim of future research would be to understand how students cope with the contextual stressors in PE identified in this case study. Research exploring the similarities and differences between genders and personal factors related to coping strategies would add even more knowledge to the field.

## Conclusions

This case study reveals nuances identified using different qualitative approaches and a supplementary survey. It highlights 9th-grade students' multitude of perceived stressors experienced during PE lessons, including stressors related to the teaching, physical, and social environments, as well as personal factors. The *empirical implications* from our study are that observations, individual teacher and student interviews, student focus group interviews, and a survey all point to the necessity of a positive and safe social environment with good relations, in line with a mastery-oriented climate (Nicholls, 1989; Ames, 1992). Girls generally score higher on PE-related stress than boys. Our findings shed light on certain similarities and differences that may exist between students of different genders and obtained grades and with past physical activity experiences. However, between observations, focus groups and interviews, we found both convergence, inconsistency, and contradictory findings (Mathison, 1988) regarding self-perceived stress from PE. In the final part of the study we were able to detect gender differences more concretely. The survey data shows that girls are the more vulnerable gender to contextual stressors, generally scoring higher on PE-related stress than boys. The analyses show that spectators, in addition to difficult tasks and low self-efficacy, seem particularly stressful for girls. The *methodological implications* from our case study are that method triangulation seems to be a fruitful approach to understanding stressors in PE to minimize the most common validity threats in fieldwork: researcher bias and reactivity. More specifically, combining mainly qualitative data with some survey-data conducted in the final stage of a cumulative data analysis process, gave us an opportunity to identify and check for diversity vs. uniformity in our data material (in order to avoid eventually biases overlooked earlier in the data analysis process). Such check for supporting evidence as well as negative evidence increased the internal generalizability (Maxwell, 2010) between participants and methods as a whole in our case study. Overall, our results support and expand previous research and highlight the volume and variety of potential stressors in PE contexts. The findings shed light on a certain need to conduct more large-scale studies to expand the current state of knowledge as well as the need to establish more sustainable theoretical frameworks within the research area in the coming years.

## Data Availability Statement

The datasets generated for this study are available on request to the corresponding author.

## Ethics Statement

The studies involving human participants were reviewed and approved by NSD—Norwegian Center for Research Data. Written informed consent to participate in this study was provided by the participants' legal guardian/next of kin.

## Author Contributions

E-KÅ: this article is a part of her doctoral thesis. She is the in charge for all process, research idea, design of the work, generating data, analysis and interpretation of the data, main writer of the manuscript, and provided ethical approval for publication of the content. HL: substantial contributions to the design of the work, generating data in focus groups, analysis and interpretation of the qualitative data, drafted and revising it critically for important intellectual content, and provided important contributions to the final manuscript. RK: substantial contributions to the design of the work, collected quantitative data and analyzing it, drafted and revising it critically for important intellectual content, and provided important contributions to the final manuscript. All authors: contributed to the article and approved the submitted version.

## Conflict of Interest

The authors declare that the research was conducted in the absence of any commercial or financial relationships that could be construed as a potential conflict of interest.
