# Supplementary material for: Perceptions of Contextual Stressors in Physical Education. A Qualitative Case Study
Source: Front Sports Act Living. 2020 Oct 9;2:528979. doi: 10.3389/fspor.2020.528979 (PMC7739826; doi:10.3389/fspor.2020.528979)
Supplement: Supplementary file 1 [file Data_Sheet_1.pdf]

Supplements

TABLE 1 | Information about the observations.

Class	Students	Teacher	Date	Duration	Context	Content
Mixed class 1	19 (11 M, 8 F)	Tim	March 2019	55 min	Gymnasium 1/2	Volleyball
Mixed class 2	19 (12 M, 7 F)	Tim	March 2019	60 min	Gymnasium 1/2	Volleyball
Mixed class 1	21 (9 M, 12 F)	Kane	March 2019	55 min	Gymnasium 1/2	Dance
Mixed class 2	18 (8 M, 10 F)	Kane	March 2019	60 min	Gymnasium 1/2	Dance
Class C	23 (11 M, 12 F)	Tim	March 2019	55 min	Swimming pool	Swimming
Mixed class 1	19 (9 M, 10 F)	Tim	April 2019	60 min	Gymnasium	Handball
Mixed class 2	18 (10 M, 8 F)	Kane	April 2019	60 min	Soccer field	Outdoor activity, play, relay games, softball

M: male, F: female.

TABLE 2 | Information about individual student interviews.

Student	Gender	Activity level	Duration	Prior sport experience
Jon	Male	High	27:28	Soccer, alpine skiing
Sarah	Female	?	33:34	Soccer, handball, gymnastics, athletics, horseback riding
David	Male	High	33:35	Soccer, hiking
Elsa	Female	High	26:47	Soccer, motor cross, hiking, jogging
Gina	Female	Inactive	23:22	Handball
James	Male	High	36:23	Swimming, jogging
Lewi	Male	Moderate	33:53	Jogging, hiking
Sandra	Female	Low	29:32	None
Sue	Female	Moderate	35:59	Handball, gymnastics
Sam	Male	High	23:16	Alpine skiing, soccer
Pete	Male	Inactive	24:31	Soccer, handball
Jill	Female	Low	21:13	Horseback riding, drill
Jo	Female	Moderate	23:59	Soccer

Activity level = times per week (exclusive duration and intensity of training); high: ≥5, moderate: 3–4, low: 1–2, inactive: <1, ?: not reported.

TABLE 3 | Information about individual interviews with PE teachers.

Name	Gender	Age	Seniority	Duration
Tim	Male	33	10 years	50:49
Kane	Male	33	6 years	43:04

TABLE 4 | Information about focus group interviews.

Focus group	Duration	Name	Gender	Physical activity in leisure time
**1**	00:39:48	Steve	Male	None
		Ingmar	Male	None
		Oliver	Male	Athletics
		Andrew	Male	Athletics
**2**	01:06:59	Lucy	Female	Horseback riding, swimming
		Ally	Female	Soccer, handball
		Isabell	Female	Soccer, handball
		Evie	Female	Horseback riding
**3**	01:00:46	Sophia	Female	Soccer, handball
		Daisy	Female	Soccer
		Mason	Male	Handball
**4**	00:48:34	Ariana	Female	Handball
		Anna	Female	Dance
		Jaxon	Male	Soccer, jogging

		Ian	Male	Soccer, cross-country skiing, uphill running
**5**	00:14:05[a]	Tyler	Male	Parkour
		Mateo	Male	Parkour
		Freddie	Male	Soccer

[a]Short because of recess and student's late arrival.
